# Preoperative detection of extraprostatic tumor extension in patients with primary prostate cancer utilizing [^68^Ga]Ga-PSMA-11 PET/MRI

**DOI:** 10.1186/s13244-024-01876-5

**Published:** 2024-12-12

**Authors:** Clemens P. Spielvogel, Jing Ning, Kilian Kluge, David Haberl, Gabriel Wasinger, Josef Yu, Holger Einspieler, Laszlo Papp, Bernhard Grubmüller, Shahrokh F. Shariat, Pascal A. T. Baltzer, Paola Clauser, Markus Hartenbach, Lukas Kenner, Marcus Hacker, Alexander R. Haug, Sazan Rasul

**Affiliations:** 1 Department of Biomedical Imaging and Image-guided Therapy, Division of Nuclear Medicine, Medical University of Vienna, Vienna, Austria; 2 Christian Doppler Laboratory for Applied Metabolomics, Vienna, Austria; 3 Department of Pathology, Medical University of Vienna, Vienna, Austria; 4 Center for Medical Physics and Biomedical Engineering, Medical University of Vienna, Vienna, Austria; 5 Department of Urology and Andrology, University Hospital Krems, Krems, Austria; 6 Karl Landsteiner University of Health Sciences, Krems, Austria; 7 Department of Urology, Medical University of Vienna, Vienna, Austria; 8 Department of Urology, University of Texas Southwestern Medical Center, Dallas, USA; 9 Division of Urology, Department of Special Surgery, The University of Jordan, Amman, Jordan; 10 Department of Urology, Second Faculty of Medicine, Charles University, Prague, Czech Republic; 11 Department of Urology, Weill Cornell Medical College, New York, USA; 12 Karl Landsteiner Institute of Urology and Andrology, Vienna, Austria; 13 Department of Biomedical Imaging and Image-Guided Therapy, Division of General and Pediatric Radiology, Medical University of Vienna, Vienna, Austria; 14 Center for Biomarker Research in Medicine, Graz, Austria; 15 Unit for Pathology of Laboratory Animals, University of Veterinary Medicine Vienna, Vienna, Austria

**Keywords:** Prostate cancer, PSMA, PET/MRI, Machine learning, Extraprostatic extension

## Abstract

**Objectives:**

Radical prostatectomy (RP) is a common intervention in patients with localized prostate cancer (PCa), with nerve-sparing RP recommended to reduce adverse effects on patient quality of life. Accurate pre-operative detection of extraprostatic extension (EPE) remains challenging, often leading to the application of suboptimal treatment. The aim of this study was to enhance pre-operative EPE detection through multimodal data integration using explainable machine learning (ML).

**Methods:**

Patients with newly diagnosed PCa who underwent [^68^Ga]Ga-PSMA-11 PET/MRI and subsequent RP were recruited retrospectively from two time ranges for training, cross-validation, and independent validation. The presence of EPE was measured from post-surgical histopathology and predicted using ML and pre-operative parameters, including PET/MRI-derived features, blood-based markers, histology-derived parameters, and demographic parameters. ML models were subsequently compared with conventional PET/MRI-based image readings.

**Results:**

The study involved 107 patients, 59 (55%) of whom were affected by EPE according to postoperative findings for the initial training and cross-validation. The ML models demonstrated superior diagnostic performance over conventional PET/MRI image readings, with the explainable boosting machine model achieving an AUC of 0.88 (95% CI 0.87–0.89) during cross-validation and an AUC of 0.88 (95% CI 0.75–0.97) during independent validation. The ML approach integrating invasive features demonstrated better predictive capabilities for EPE compared to visual clinical read-outs (Cross-validation AUC 0.88 versus 0.71, *p* = 0.02).

**Conclusion:**

ML based on routinely acquired clinical data can significantly improve the pre-operative detection of EPE in PCa patients, potentially enabling more accurate clinical staging and decision-making, thereby improving patient outcomes.

**Critical relevance statement:**

This study demonstrates that integrating multimodal data with machine learning significantly improves the pre-operative detection of extraprostatic extension in prostate cancer patients, outperforming conventional imaging methods and potentially leading to more accurate clinical staging and better treatment decisions.

**Key Points:**

- Extraprostatic extension is an important indicator guiding treatment approaches.
- Current assessment of extraprostatic extension is difficult and lacks accuracy.
- Machine learning improves detection of extraprostatic extension using PSMA-PET/MRI and histopathology.

**Graphical Abstract:**

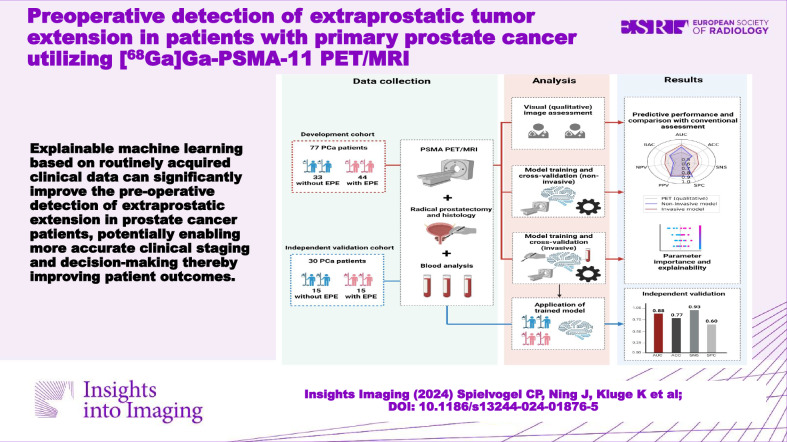

## Introduction

Radical prostatectomy (RP) is a common treatment option for patients with localized prostate cancer (PCa) [1]. While RP often provides excellent curative results, functional adverse effects such as erectile dysfunction associated with surgical destruction of the neurovascular bundles can occur [2]. In order to balance postoperative functional [3] and long-term disease outcomes [4], current practice guidelines recommend, therefore, nerve-sparing RP in case of localized tumors. For tumors with extraprostatic extension (EPE), including tumors with extracapsular extension (ECE) (T3a) or seminal vesicle invasion (SVI) (T3b), wide resection of periprostatic tissue is recommended [1].

However, accurate pre-operative detection of EPE is challenging due to the suboptimal sensitivity of multiparametric magnetic resonance imaging (mpMRI) and positron emission tomography (PET) radiotracers that target prostate-specific membrane antigen (PSMA), which often leads to inadequate selection of surgical extent [5, 6]. Therefore, a more accurate approach for the pre-operative assessment of EPE is highly desirable.

Machine learning (ML) can potentially enable more accurate assessment by multimodal integration of imaging and clinical data. However, two of the main criticisms of clinical ML applications are the lack of insight into their decision-making procedures and the fact that there are few specialists who are skilled in both clinical and ML aspects. On the one hand, novel explanatory methods can improve validation, risk assessment, safety, comprehensibility and the integration of ML systems with previously derived clinical knowledge, leading to greater acceptance in the medical community and thus to better health outcomes [7–9]. On the other hand, automated ML approaches promise to partially lift restrictions in terms of the ML expertise required for clinical applications.

In this work, we therefore employ explainable and automated ML to non-invasively and pre-operatively predict the presence of EPE in men with primary PCa based on PSMA-PET/MRI, histological, and clinical parameters to explore its potential for explainable ML-assisted surgical guidance.

## Materials and methods

### Study design

**Fig. 1:**
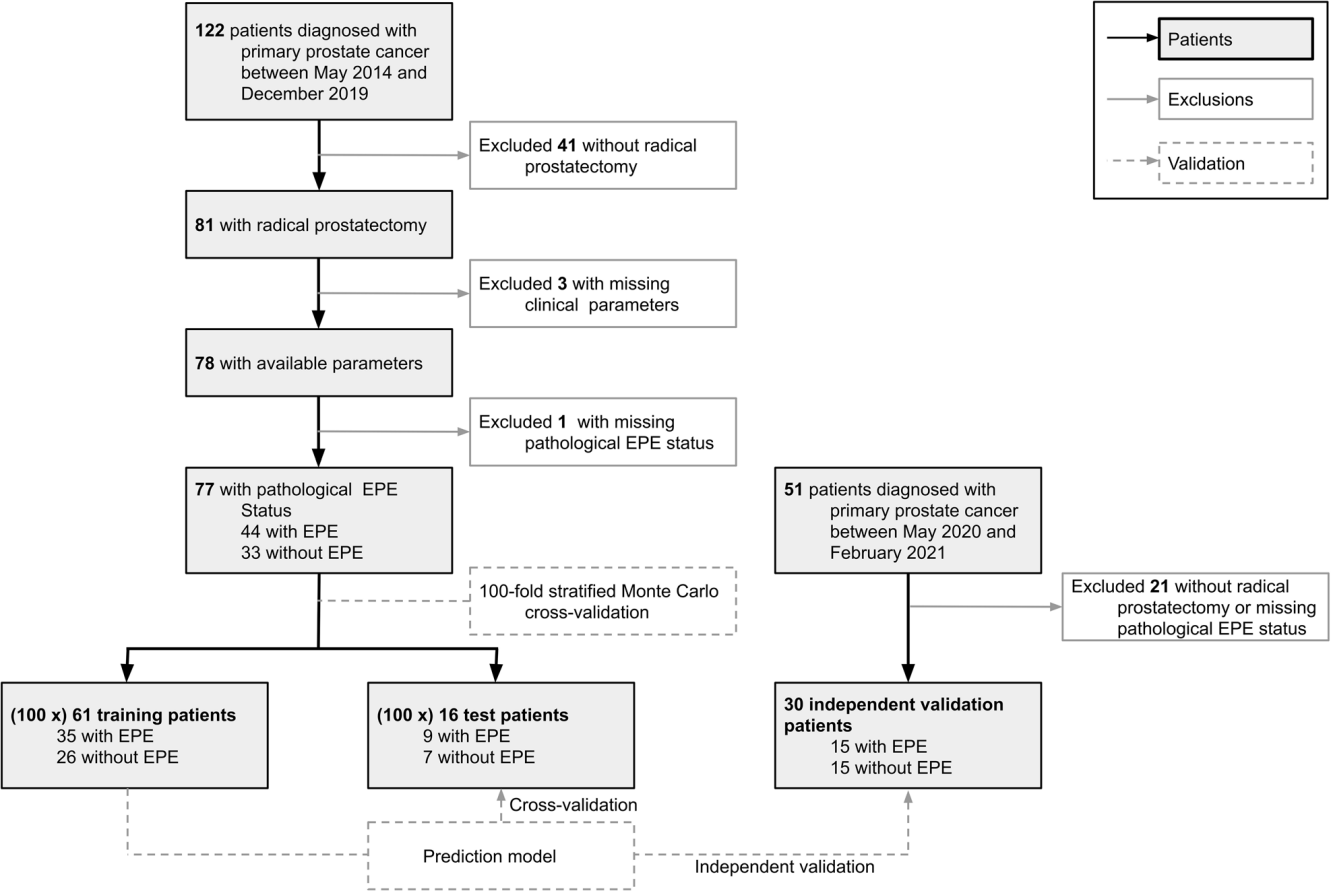
Cohort flow and validation diagram. A total of 107 patients were included in the overall analysis, 77 of whom were in the development cohort (training and cross-validation) and 30 who were part of the independent validation cohort. EPE, extraprostatic extension

For this pilot study of a prospective registered clinical trial (NCT02659527), patients who underwent RP and received a pre-operative [^68^Ga]Ga-PSMA-11 PET/MRI scan between May 2014 and December 2019 were retrospectively enrolled to be employed during the development (training and cross-validation) phase. For independent validation, patients with the same inclusion and exclusion criteria were enrolled who received a [^68^Ga]Ga-PSMA-11 PET/MRI scan between May 2020 and February 2021. Exclusion criteria are shown in the cohort flow diagram (Fig. 1).

All patients underwent [^68^Ga]Ga-PSMA-11 PET/MRI scans and received RP within a median of 43 ± 26 days after the scan. In total, 24 pre-operative parameters were extracted for each patient from the clinical data archive of the Vienna General Hospital. Eleven qualitative, visually assessed PET/MRI features were determined as part of the clinical routine. In addition, six semi-quantitative PET/MRI features as well as two blood-derived parameters (pre-operative PSA and serum testosterone levels), three biopsy-derived parameters including International Society of Urological Pathology (ISUP) grading, ratio of tumor to overall tissue and ratio of tumor-containing biopsy samples, and two general patient characteristics including age and body mass index (BMI) were acquired. Criteria for the selection of the employed parameters included their routine assessment to ensure seamless integration of the developed model into the clinical workflow, agreement with the parameters acquired as specified by the study protocol of the associated clinical trial (NCT02659527), and a high probability for robustness to center-effects, ensuring robustness and generalizability beyond the development cohort.

**Fig. 2:**
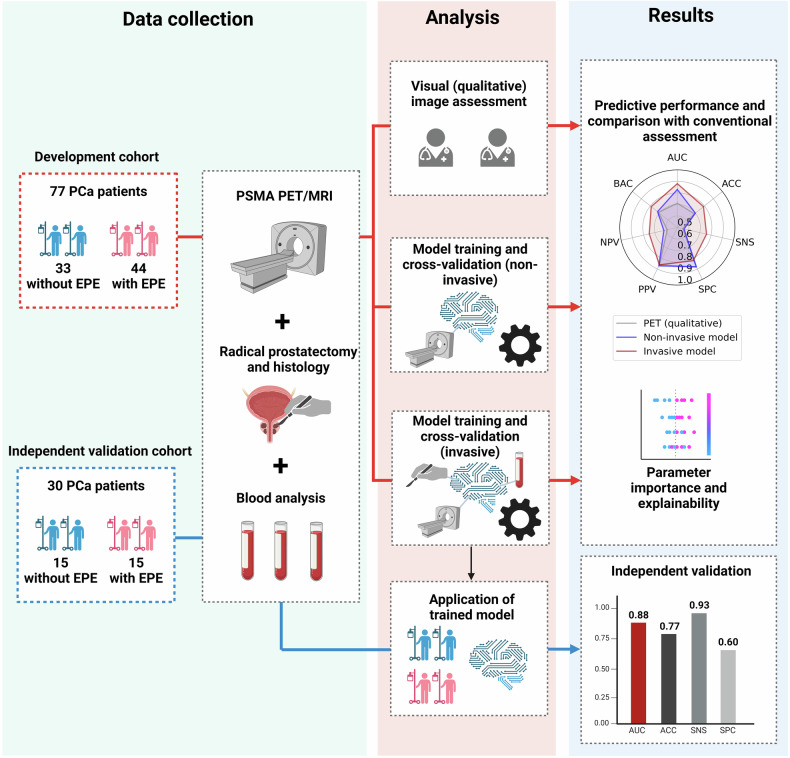
Study workflow. A total of 77 patients with localized prostate cancer (PCa) underwent PET/CT examination and radical prostatectomy. Three approaches for the assessment of extraprostatic extension were compared: (1) Visual qualitative assessment as performed in clinical routine, (2) Machine learning modeling using non-invasive parameters, and (3) Machine learning modeling using invasive and non-invasive parameters. Comparative diagnostic performance was assessed, and explainability methods for the machine learning models were integrated. The model using invasive and non-invasive parameters was then validated on an independently collected cohort of additional 30 PCa patients

The prediction target was EPE, which was defined as the presence of either SVI or ECE based on the post-surgical histopathology results. Two ML-based classification models were created for the prediction of EPE. One model was based on all 24 pre-operative features while the other model was based only on non-invasively acquired parameters (exclusion of histology and blood parameters). The diagnostic performance of the ML models was compared to the pre-operative PET/MRI readings. An overview of the study workflow is shown in Fig. 2. The study was conducted with adherence to the Declaration of Helsinki and approved by the local ethics committee (ethics ID: EK 1985/2014). Patients approved their participation via written informed consent. Reporting was performed in accordance with the CLAIM guidelines [10].

### Image reading

[^68^Ga]Ga-PSMA-11 PET/MRI images were interpreted using the Hermes Hybrid 3D software (Hermes Medical Solutions, Sweden) by two clinical experts in hybrid imaging (M. Hartenbach, nuclear medicine; P. Baltzer, radiology). PET/MRI-derived features included tumor stage, nodule status, metastasis stage, presence of bone metastases, neurovascular bundle invasion, capsule penetration, whether the prostate apex or whether the prostate base was contacted by the tumor, seminal vesicle invasion, presence of EPE, and the semi-quantitative maximum standardized uptake value (SUVmax), SUVmean, SUVmin, SUVpeak, total lesion PSMA (TL-PSMA), and PSMA-derived tumor volume (PSMA-TV). Grading and staging were performed in accordance with the Union Internationale Contre Le Cancer (UICC) tumor node metastasis (TNM) classification of 2009 [11]. EPE was determined to be present if either MRI or PSMA-PET findings were suggestive of EPE. On MRI, visual assessment of EPE was performed as suggested by Bloch et al [12]. For PET, EPE status was positive if high PSMA uptake was present outside the prostate capsule, especially in the areas of the neurovascular bundles and surrounding tissue. Details on image acquisition can be found in the Supplementary section “Imaging protocol”.

### Machine learning

To ensure robust performance estimation, 100-fold stratified Monte Carlo cross-validation was performed with 80% of samples randomly assigned to the training set and 20% of samples assigned to the test set as part of the initial training and cross-validation phase. The best model during cross-validation was selected and further validated on an independent test set. A total of five classification algorithms were used, including decision tree (DT), logistic regression (LGR), random forest (RF), extreme gradient boosting (XGB), and explainable boosting machine (EBM) [13]. Evaluation performance metrics included area under the receiver operating characteristic curve (AUC), accuracy, balanced accuracy, sensitivity, specificity, positive predictive value and negative predictive value. Metric formulas are shown in the Supplementary section “Performance metrics”. Details on preprocessing are shown in the Supplementary section “Machine learning preprocessing”.

Next to inherently interpretable models such as logistic regression and EBM classifier, a variety of explainable artificial intelligence functionalities were employed. Insights into the predictive importance and directionality of the importance of individual features were investigated via Shapley additive explanations (SHAP) [14]. EBM-specific global feature-wise partial dependence plots (PDP) were created to indicate the precise change in model output with respect to feature values. Exploratory data analysis was performed, including univariate descriptive statistics for each feature and dimensionality reduction based on uniform manifold approximation and projection (UMAP) [15].

### Statistical analysis

Continuous data were reported as mean ± standard deviation (SD) or median and interquartile range. Categorical variables were expressed as numbers and percentages. Diagnostic performance metrics are provided in association with 95% confidence intervals (CI), calculated over cross-validation folds where possible. For the independent validation, 95% confidence intervals were calculated using 10,000 bootstrapping samples. Any missing values were imputed using k-nearest neighbor imputation. Subgroup differences in patient characteristics were assessed using *t*-tests or Mann–Whitney U tests for continuously distributed variables and either Fisher’s exact test or Chi-squared test for categorical variables. AUC values were compared using the DeLong test. Statistical significance was defined as *p*-values ≤ 0.05. All employed software is listed in the Supplementary section “Software”.

## Results

### Patients

For the development cohort, 122 patients with localized PCa were assessed, 45 of whom had to be excluded due to reasons displayed in Fig. 1. A total of 77 patients were eligible for the initial training and cross-validation procedure. Patient characteristics stratified with respect to the presence of EPE are shown in Table 1. Among the 77 patients, 44 (57%) were diagnosed with EPE based on the post-surgical histology results, with 28 (36%) patients affected by EPE based on visual PET/MRI assessment. Interactivate descriptive statistics of the entire data can be accessed via https://cspielvogel.github.io/epe_descriptive_statistics/epe_descriptive_statistics.html. Dimensionality reduction via UMAP indicated a tendency toward similarity of patients with EPE over all features (Supplementary Fig. 1). An independent validation cohort was collected after the model development procedure was concluded. The independent validation cohort was comprised of 30 patients with 15 (50%) being affected by EPE based on histology. The patient characteristics of the independent validation cohort are displayed in Supplementary Table 3.

**Table 1:** Patient characteristics including stratification by histology-confirmed extraprostatic extension status

Parameter	Value	Entire data	No EPE	EPE	*p*-value
Number of patients, *n* (%)		77 (100.00%)	33 (42.86%)	44 (57.14%)	
Age		64.97 (7.16)	63.85 (7.7)	65.82 (6.6)	0.2813
BMI		27.06 (3.51)	27.03 (3.45)	27.09 (3.55)	0.9464
ISUP					
	1	16 (20.78%)	12 (36.36%)	4 (9.09%)	< 0.0001*
	2	17 (22.08%)	12 (36.36%)	5 (11.36%)	
	3	14 (18.18%)	1 (3.03%)	13 (29.55%)	
	4	16 (20.78%)	4 (12.12%)	12 (27.27%)	
	5	8 (10.39%)	0 (0%)	8 (18.18%)	
	NA	6 (7.79%)	4 (12.12%)	2 (4.55%)	
Neurovascular bundle contact (PET/MRI)		9 (11.69%)	1 (3.03%)	8 (18.18%)	0.0697
Bone metastases (PET/MRI)		5 (6.49%)	0 (0%)	5 (11.36%)	0.0670
Nodule status (PET/MRI)		11 (14.29%)	0 (0%)	11 (25.0%)	0.0018*
Metastases (PET/MRI)		7 (9.09%)	0 (0%)	7 (15.91%)	0.0177*
Location both sides (PET/MRI)		17 (22.08%)	8 (24.24%)	9 (20.45%)	0.7839
Location base (PET/MRI)		15 (19.48%)	3 (9.09%)	12 (27.27%)	0.0709
Location apex (PET/MRI)		0.38 (0.48)	0.33 (0.47)	0.41 (0.49)	0.5039
Seminal vesicle invasion (PET/MRI)					
	Yes	19 (24.68%)	0 (0%)	19 (43.18%)	< 0.0001*
	NA	2 (2.6%)	0 (0%)	2 (4.55%)	
Extracapsular extension (PET/MRI)	0	26 (33.77%)	4 (12.12%)	22 (50.0%)	0.0006*
Extraprostatic extension (PET/MRI)	0	28 (36.36%)	4 (12.12%)	24 (54.55%)	0.0001*
Tumor stage (PET/MRI)					
	cT2a	13 (16.88%)	7 (21.21%)	6 (13.64%)	< 0.0001*
	cT2b	9 (11.69%)	4 (12.12%)	5 (11.36%)	
	cT2c	22 (28.57%)	18 (54.55%)	4 (9.09%)	
	cT3a	12 (15.58%)	4 (12.12%)	8 (18.18%)	
	cT3b	14 (18.18%)	0 (0%)	14 (31.82%)	
	cT3a+b	5 (6.49%)	0 (0%)	5 (11.36%)	
	cT4	2 (2.6%)	0 (0%)	2 (4.55%)	
Ratio positive cylinders		0.45 (0.32)	0.26 (0.22)	0.62 (0.29)	< 0.0001*
Positive tissue ratio		0.37 (0.25)	0.29 (0.18)	0.45 (0.28)	0.0342*
Pre-operative PSA		34.5 (97.38)	10.89 (8.79)	52.2 (125.72)	0.0112*
Pre-operative testosterone		3.44 (2.19)	4.08 (1.83)	3.1 (2.28)	0.2173
SUVpeak		9.82 (7.36)	7.63 (3.52)	11.46 (8.91)	0.0362*
SUVmean		6.84 (4.88)	5.5 (2.25)	7.84 (5.96)	0.0750
SUVmin		3.18 (3.47)	2.76 (1.46)	3.51 (4.38)	0.3577
SUVmax		14.35 (11.09)	11.27 (6.81)	16.66 (12.95)	0.0443*
TL-PSMA		67.07 (73.33)	47.26 (52.28)	81.93 (82.73)	0.0395*
PSMA-TV		9.41 (8.18)	8.95 (10.21)	9.76 (6.22)	0.1527

Data are *n*, mean (SD), or *n* (% of patients). Asterisks indicate significance

*EPE* extrapostatic extension, *BMI* body mass index, *ISUP* International Society of Urological Pathology, *PSA* prostate-specific antigen, *NA* not applicable (unknown)

### Diagnostic performance of machine learning models

**Fig. 3:**
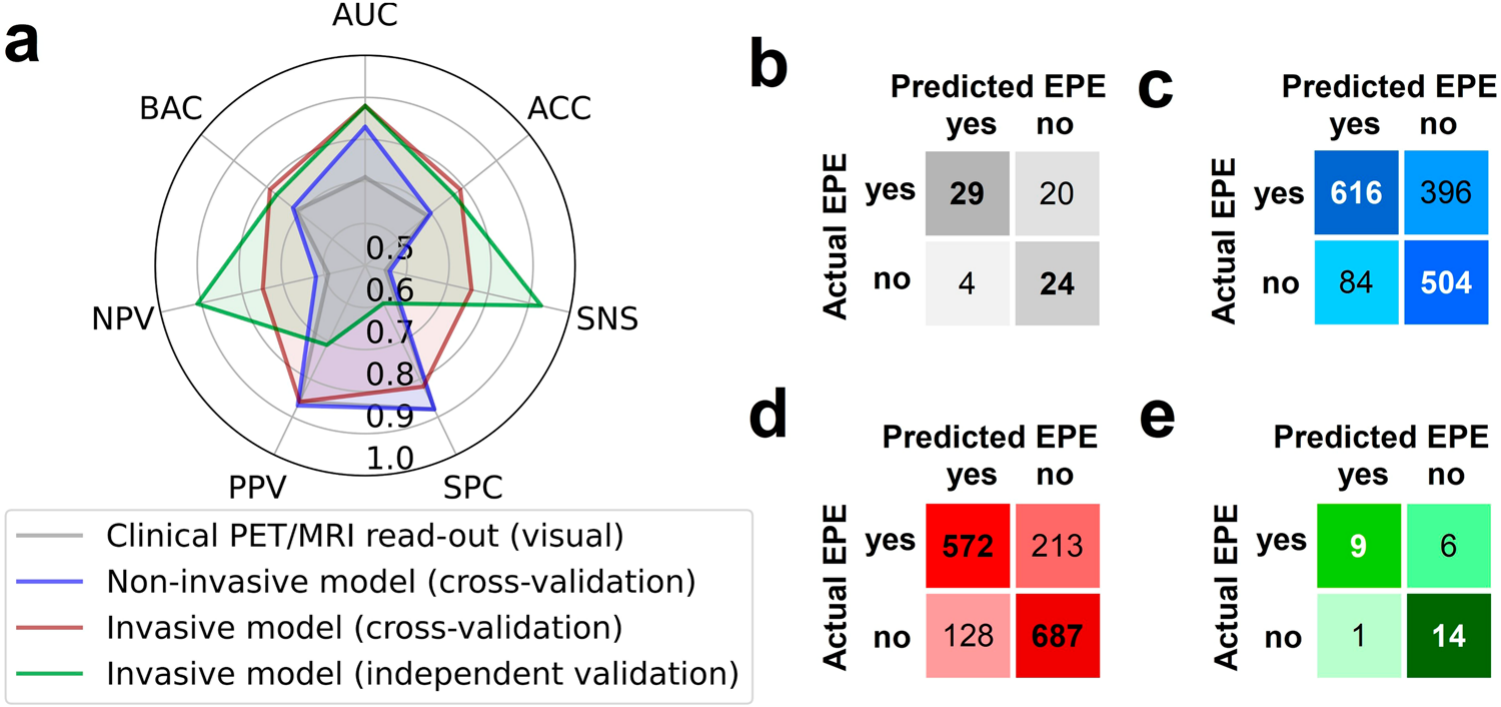
Comparison of the three investigated approaches for pre-operative EPE detection. **a** Diagnostic performance metrics of the three approaches. **b** Confusion matrix of the visual clinical PET/MRI read-out. **c** Confusion matrix for the machine learning model based on non-invasive features only. **d** Confusion matrix for the machine learning model based on non-invasive and invasive features. **e** Confusion matrix for the machine learning model with non-invasive and invasive features on the independent test cohort. For **c** and **d**, predictions are shown cumulatively over all 100 folds. ACC, accuracy; SNS, sensitivity; SPC, specificity; PPV, positive predictive value; NPV, negative predictive; BAC, balanced accuracy; AUC, area under the receiver operating characteristic curve; EPE, extraprostatic extension

For the prediction of EPE using all available pre-operative features, the highest 100-fold Monte Carlo cross-validation AUC was associated with the EBM at AUC 0.88 (95% CI 0.87–0.89), sensitivity 0.76 (95% CI 0.73–0.79), specificity 0.82 (95% CI 0.79–0.85), positive predictive value 0.86 (95% CI 0.84, 0.88), negative predictive value 0.75 (95% CI 0.73–0.77), balanced accuracy 0.79 (95% CI 0.77–0.81), and accuracy 0.79 (95% CI 0.77–0.81). For the second model built using only non-invasive features, the EBM was again the best-performing classifier with AUC 0.83 (95% CI 0.81–0.85), sensitivity 0.56 (95% CI 0.53–0.59), specificity 0.88 (95% CI 0.85–0.91), positive predictive value of 0.88 (0.86–0.90), negative predictive value of 0.62 (95% CI 0.60–0.64), balanced accuracy of 0.72 (95% CI 0.70–0.74), and accuracy of 0.70 (95% CI 0.68–0.72). Since the EBM surpassed the performance of standard clinical PET/MRI read-out, an additional independent validation was performed to confirm the findings. In this additional validation step, the EBM achieved an AUC of 0.88 (95% CI 0.75–0.97), sensitivity 0.93 (95% CI 0.80–1.00), specificity 0.60 (95% CI 0.33–0.87), positive predictive value 0.71 (95% CI 0.58, 0.87), negative predictive value 0.71 (95% CI 0.71–1.00), balanced accuracy 0.77 (95% CI 0.63–0.90), and accuracy 0.77 (95% CI 0.63–0.90). The full spectrum of performance metrics is shown in Fig. 3a. The performance of other classifiers during cross-validation is shown in Supplementary Figs. 2 and 3.

### Comparison with visual PET/MRI interpretation

To introduce a baseline model for comparison with ML-derived results, clinical-standard visual PET/MRI-based pre-operative EPE estimation was compared to the histopathological ground truth to derive diagnostic performance metrics. Visual interpretation yielded an AUC of 0.71, sensitivity 0.55, specificity 0.88, positive predictive value 0.86, negative predictive value 0.59, balanced accuracy 0.71, and accuracy of 0.69. Performance metrics and confusion matrices are shown in Fig. 3. Comparison of AUCs via DeLong test indicated a significant improvement of the ML model with invasive over non-invasive features only (*p* < 0.0001). Further, there was a significant difference between the ML model with invasive features and the visual clinical PET/MRI read-out (*p* = 0.02). However, there was no difference between the ML model with non-invasive features only and the visual clinical PET/MRI read-out (*p* = 0.34).

### Explainability of machine learning models

**Fig. 4:**
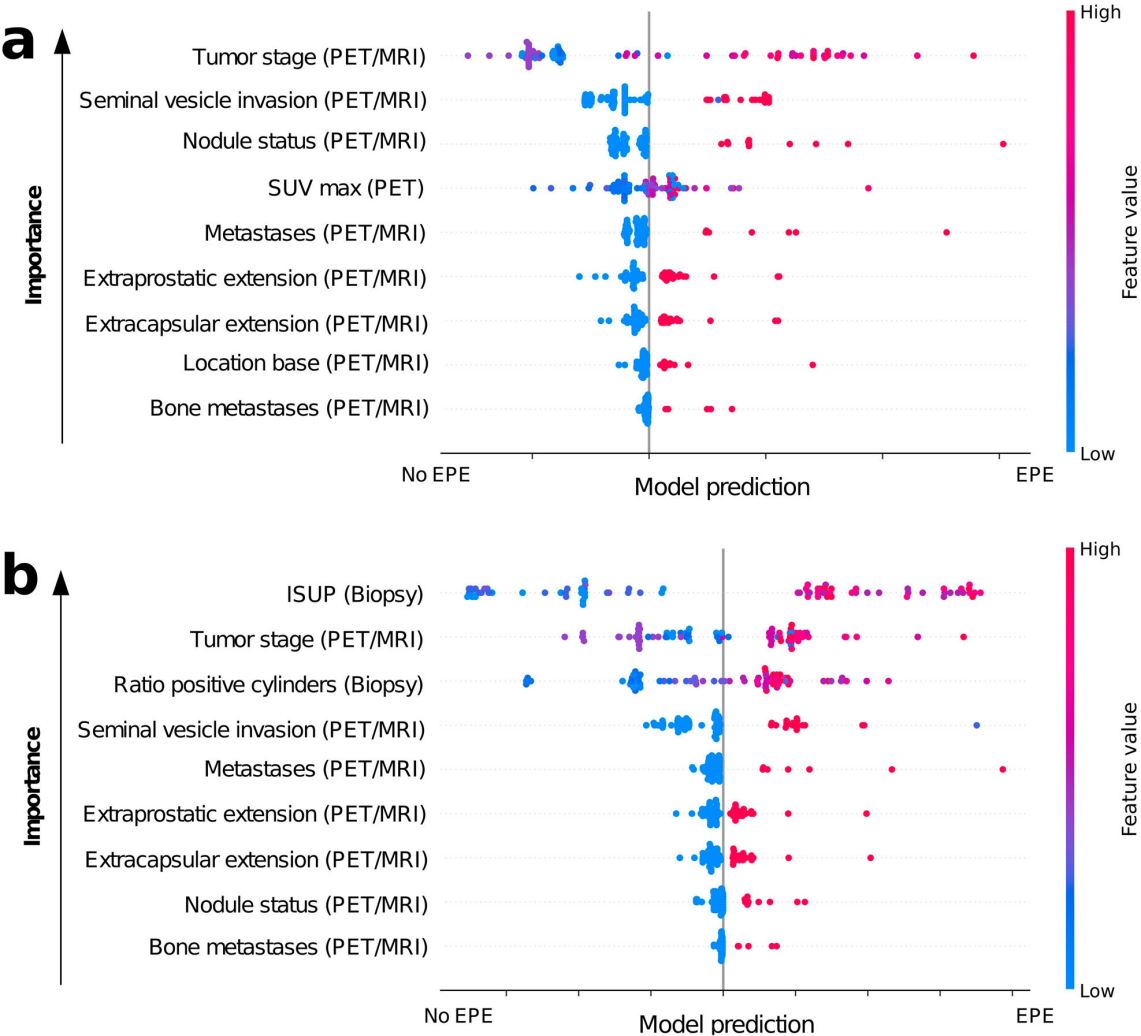
Global SHAP feature importance for the machine learning models with (**a**) non-invasive features only and (**b**) for all features. Features are ranked in order of their importance with the most important feature at the top. Each dot represents a single patient’s prediction. Colors indicate the magnitude of the feature value in each row for the given patient. The *x*-axis indicates the model’s tendency for the prediction of EPE or no-EPE due to the given feature value for the given patient. ISUP, International Society of Urological Pathology (grade); EPE, extraprostatic extension

For the ML model, including invasive features, ISUP and the ratio of positive biopsy cylinders were ranked most and third most important in the feature importance estimation based on SHAP (Fig. 4). Apart from the addition of the two invasively derived features, the remaining non-invasive features were approximately consistently important over the invasive and purely non-invasive model. A notable exception was SUVmax, which ranked fourth most important in the non-invasive model but was missing from the model with invasive features. Owing to the discrepancy observed in the importance of SUVmax across the two models, we hypothesized that SUVmax might be redundant, and potentially correlated with one of the invasive features. As a result, the ML algorithm might have excluded the feature from the model. Hence, we tested for linear correlation between SUVmax and other features (Supplementary Fig. 4). Spearman rank test indicated a slight but monotonous and highly significant correlation between ISUP grade and SUVmax (*R* = 0.37, *p* < 0.001, Supplementary Fig. 8). Due to differences in feature importance estimation methods, we validated SHAP importance using permutation importance estimations (Supplementary Figs. 5 and  6) which confirmed the SHAP feature importance despite expected minor variations. Since SHAP feature importance has a low resolution in terms of providing insights into the association of precise feature values and the model output, EBM-specific PDPs were created (Supplementary Fig. 7). The PDPs confirmed the expected feature directions indicated by the SHAP analysis, however, indicated a counter-intuitive, slight increase of the probability of the model outputting a positive EPE status at tumor stage cT2c compared to lower stages.

## Discussion

In this pilot study of the registered clinical trial (NCT02659527), EPE was successfully predicted from routinely recorded pre-operative parameters. The diagnostic performance of the explainable ML models was compared to the performance of visual clinical read-outs of [^68^Ga]Ga-PSMA-11 PET/MRI imaging. The ML models increased the diagnostic performance by AUC 0.17 (0.71 vs 0.88), sensitivity 0.21 (0.55 vs. 0.76), negative predictive value 0.16 (0.59 vs. 0.75), balanced accuracy 0.08 (0.71 vs. 0.79) and accuracy 0.17 (0.71 vs. 0.88) while the positive predictive value was the same and specificity was slightly higher for the visual assessment (0.88 vs. 0.82). The model, which was based purely on non-invasive features, was slightly better compared to clinical read-outs; however, the difference was not significant. In the independent validation, the performance of the invasive model remained high (AUC 0.88), indicating the robustness of the model.

We performed a literature search using PubMed on December 4th, 2023, with the following search query: (“machine learning” OR “deep learning” OR “artificial intelligence”) AND PET AND (“extracapsular extension” OR “extraprostatic extension”). The search yielded a total of three publications [16–18], all of which were original articles published between February 2021 and November 2023. In each of these three investigations, the predictive analysis was exclusively based on imaging characteristics. These encompassed both experimental radiomics features and standard SUV parameters. However, none of these studies incorporated blood, demographic, or histological factors in their predictive models. Imaging was performed in all three publications using PET/CT rather than PET/MRI, and only the PET modality was considered for feature extraction. All studies employed Flourine-18 labeled PSMA (either [^18^F]DCFPyL or [^18^F]F-PSMA-1007) instead of the here employed [^68^Ga]Ga-PSMA-11 and predicted ECE instead of EPE. The performances of the associated studies ranged from AUC 0.63 to 0.77.

The 2024 EAU guidelines have only issued a weak recommendation for MRI to detect EPE due to its insufficient sensitivity to identify all tumors with extraprostatic growth and its reliance on interpretive expertise, affecting diagnostic consistency [5]. The advent of PSMA-PET/MR, as applied in this study, offers enhanced sensitivity and specificity by targeting molecular features of prostate cancer. Overall, our findings contribute to the limited but growing evidence supporting multimodal approaches, enhancing staging accuracy by combining MRI’s spatial capabilities with PSMA-PET’s molecular precision.

Particularly in mpMRI, image quality is paramount to ensure diagnostic accuracy, especially for local staging assessments of prostate cancer patients who underwent MRI-guided biopsy [19]. High-quality MRI allows for more accurate delineation of the prostate capsule and neighboring structures, critical in EPE detection [20]. Francesco Giganti et al have demonstrated that PI-QUAL v2 simplifies image assessment and ensures that high-quality images can be used for the subsequent diagnosis [21]. Low-quality images can obscure early extraprostatic signs, increasing false negatives. Ensuring high-quality imaging protocols and standardizing MRI parameters, as well as the integration of hybrid imaging modalities such as PET/MRI can improve EPE diagnostic accuracy, further supporting the integration of advanced imaging into pre-operative assessment workflows.

In our study, we decided to omit experimental radiomics features due to the current limitations in their readiness for clinical application. Despite significant advances in the field of radiomics, considerable challenges remain that impede their integration into clinical practice. One of the primary issues is the lack of standardization, a concern that persists despite efforts such as the Image Biomarker Standardization Initiative [22]. This lack of standardization affects radiomics features, making them susceptible to variability depending on the center, scanner, and protocol used, particularly in nuclear medicine and PET applications. Moreover, radiomics features are sensitive to data formats, preprocessing, and dataset inhomogeneity, leading to challenges in reproducibility and standardization [23, 24]. Given these considerations and the fact that radiomics features have not yet been implemented in clinical settings, our opportunistic ML models allow for the integration of features solely collected as part of routine clinical practice. This approach aims to facilitate a more straightforward and reliable integration of these models into clinical workflows, ensuring safer, more interpretable, and more effective implementation in real-world clinical scenarios. Still, radiomics may represent a legitimate approach for the assessment of image-based extraprostatic extension. For example, a previously performed meta-analysis suggests that among the 13 publications analyzed, the mean pooled AUC for the detection of extraprostatic extension via MRI radiomics was 0.80 [25].

**Fig. 5:**
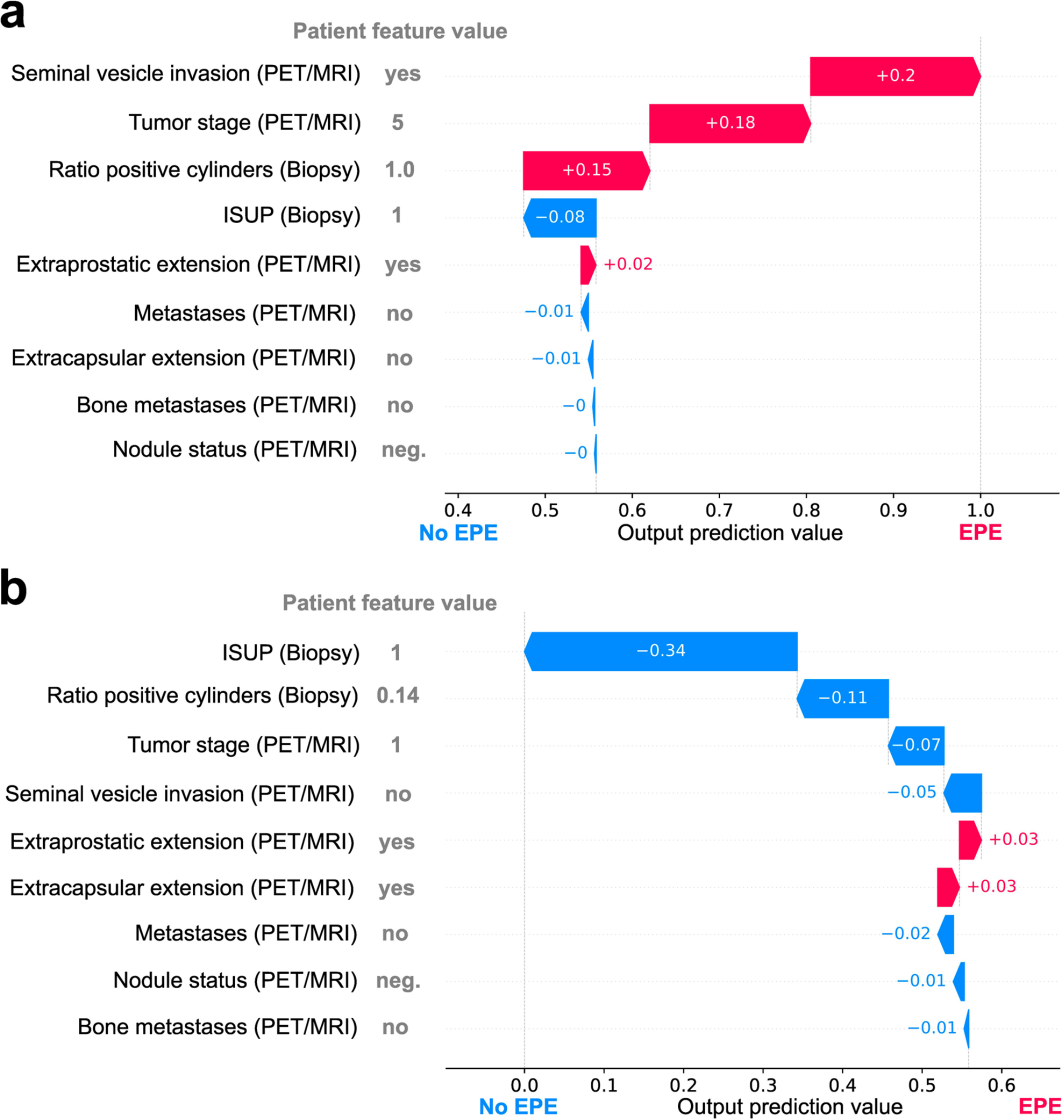
SHAP importance for two example patients correctly predicted by the non-invasive model. Blue arrows indicate a shift of the model prediction toward non-EPE due to the given feature while pink arrows indicate a shift toward a prediction of EPE. In **a**, an example patient with EPE is shown. Despite the feature with the highest importance (ISUP grade) being 1, therefore indicating a tendency toward the prediction of non-EPE, the final prediction of the model was EPE-positive since features including seminal vesicle invasion, tumor stage, and ratio of positive biopsy cylinders were in favor for the presence of EPE. In **b**, the model predicted the patient correctly as EPE-negative even though the visual PET/MRI-based assessment by the imaging physician indicated positivity for both, extraprostatic and extracapsular extension. ISUP, International Society of Urological Pathology (grade); EPE, extraprostatic extension

The interpretability of our approach was further enhanced by the application of the EBM algorithm, in which each feature of an EBM model contributes to predictions in a modular fashion. Consequently, the impact of each isolated feature at a given value can be precisely determined instead of approximation via methods such as SHAP. This allows to enable trust in the model by demonstrating that reasonable and clinically known associations are exploited by the model to make its predictions. For example, it is reasonable to assume that a higher ratio of tumor-positive biopsy cylinders is associated with a higher aggressiveness and spread of the tumor (Supplemental Fig. 7), likely being correlated with the occurrence of EPE. Furthermore, when integrating novel biomarkers into a glass-box model such as an EBM, a precise assessment of the influence of a novel biomarker on the model can be obtained. This may help to understand the role of the biomarker, allow for integration with existing medical knowledge, and enable the possibility of more precise validation of the feature robustness when employed in other models and scenarios. Our study further confirms previous findings identifying SUVmax as a non-invasive indicator for Gleason score and ISUP grade [26]. Both EBM and SHAP can further provide insights into the relative contribution of individual parameters, allowing physicians to not only better understand the predictive model but also improve the understanding of the relevance of risk factors independently of the model. Explainability methods, including EBM and SHAP, can also provide guidance on how to use a model. For example, features may not always be available in clinical practice. If a feature is missing, it may be imputed or approximated to allow for the application of the predictive model. The relative, global feature importance provided by EBM and SHAP can guide physicians in understanding how severe the impact of the missingness of a feature may be and, therefore, whether the model can be trusted in a given scenario. Additionally, SHAP can be employed to provide insights into an individual patient’s prediction, highlighting why the model assumes that a given prediction may be reasonable for a given patient. Two examples of such a personalized SHAP-based prediction for our model are shown in Fig. 5. Accurate pre-operative identification of EPE is challenging but crucial for the clinical management of PCa patients. EPE, encompassing the tumor’s local spread beyond the prostate, is detrimental for clinical staging and crucial for surgical decision-making, hormone, and radiation therapy decisions. As a consequence, our findings indicate that ML approaches may enhance TNM staging accuracy and treatment decision-making in PCa. Despite the thorough validation of our findings through explainable artificial intelligence approaches and robust cross-validation as well as independent validation, our study is not free of limitations, and the results have to be interpreted with caution. ML models employing medical imaging data are known to be sensitive to variations in imaging protocol, scanner, and center. In the present work, we aimed to counteract this phenomenon by choosing robust imaging features which are assessed as part of the clinical routine. The use of well-established features in conjunction with explainability methods further allowed us to assess whether the model learned reasonable associations between features and the prediction target, which is of high importance in this study due to the limited cohort size and single-centric nature. The primary objective of this research was to explore the integration of established parameters through ML to enhance clinical procedures. This approach aimed to offer a robust and practical solution, circumventing the need to acquire new, potentially complex, unreliable, and expensive parameters and avoiding upstream alterations to the existing clinical workflow. Hence, it was beyond the scope of this work to discover new parameters linked to EPE in primary prostate cancer. Lastly, despite the rigorous validation scheme employing both, cross-validation and an independent validation set, further validation on external cohorts is required to ensure generalizability. Despite these limitations, this study provides solid evidence for the possibility of integrating ML-driven tools for supporting surgical decision-making by investigating a well-characterized cohort with comprehensive imaging and pathological information alongside rigorously applied machine learning methods assessing the successful model training despite limits in cohort size.

In conclusion, our study demonstrates that routinely acquired parameters have the potential to aid clinical treatment decisions in prostate cancer patients via pre-operative detection of extraprostatic extension. The findings of this study suggest that employing explainable machine learning models for the anticipation of extraprostatic extension improves diagnostic performance in comparison to standard visual assessment of [^68^Ga]Ga-PSMA-11 PET/MRI with potential implications for clinical staging and treatment decision-making.

## Supplementary information

ELECTRONIC SUPPLEMENTARY MATERIAL

## Data Availability

Aggregated descriptive statistics for the employed data can be found at https://cspielvogel.github.io/epe_descriptive_statistics/epe_descriptive_statistics.html. The de-identified, raw data employed in this study is available from the corresponding author upon reasonable request.

## Abbreviations

AUC: Area under the receiver operating characteristic curve
BMI: Body mass index
EBM: Explainable boosting machine (EBM)
EPE: Extraprostatic extension
ISUP: International Society of Urological Pathology
ML: Machine learning
mpMRI: Positron-emission tomography
PCa: Prostate cancer PCa
PDP: Partial dependence plots
PSMA: Prostate-specific membrane antigen
PSMA-TV: PSMA tumor volume
RP: Radical prostatectomy
SD: Standard deviation
SHAP: Shapley additive explanations
SUV: Standardized uptake value
SVI: Seminal vesicle invasion
TL-PSMA: Total lesion PSMA
TNM: Tumor node metastasis (stage)
UMAP: Uniform manifold approximation and projection
