# Supplementary material for: Preoperative detection of extraprostatic tumor extension in patients with primary prostate cancer utilizing [68Ga]Ga-PSMA-11 PET/MRI
Source: Insights Imaging. 2024 Dec 12;15:299. doi: 10.1186/s13244-024-01876-5 (PMC11638435; doi:10.1186/s13244-024-01876-5)
Supplement: Supplementary file 1 — ELECTRONIC SUPPLEMENTARY MATERIAL [file 13244_2024_1876_MOESM1_ESM.pdf]

Preoperative detection of extraprostatic tumor extension in patients with primary prostate cancer utilizing [68Ga]Ga-PSMA-11 PET/MRI ELECTRONIC SUPPLEMENTARY MATERIAL

Imaging protocol

Every patient rested in a quiet room for 45 minutes before undergoing [^{68}Ga]Ga-PSMA-11/MRI. The tracer was injected intravenously in a standardized dose of 2 MBq/kg. The MRI contrast agent Gd-DOTA (Dotarem®, Guerbet, France), was administered intravenously at the dose of 2 ml/kg. All PET/MRI examinations were performed on a whole-body hybrid PET/MRI scanner (Biograph mMR; Siemens Healthcare, Erlangen, Germany) with a 3T MRI system. PET was performed with a 4 minutes emission scan/bed position (2 beds) for the pelvic region. The final whole body PET scan was performed with 4 bed positions, 4 minutes sinogram mode each. Reconstruction parameters for PET were: 3 iterations/ 21 subsets for static images; 1 frame/2 minutes with 3 iterations/21 subsets for listmode [^{68}Ga]Ga-PSMA-11 data as well as the summation of the last 10 minutes [^{68}Ga]Ga-PSMA-11 acquisition for visual analysis. MRI parameters are shown in **Supplementary Table 1**.

Machine learning preprocessing

Preprocessing was performed for each fold separately to avoid data leakage. Fold-wise preprocessing was conducted including feature standardization, feature imputation via k-nearest neighbor imputation, feature selection via the minimum redundancy maximum relevance (mRMR) algorithm [1], imbalance handling via the synthetic minority oversampling technique (SMOTE) [2] and an automated hyperparameter optimization via random search through a reasonable parameter grid (**Supplementary Table 2**).

Software

Python 3.9.5 was used for all analyses and visualizations. The following Python packages were employed: Numpy [3], Pandas [4], Seaborn [5], Matplotlib [6], Scikit-learn [7], Imbalanced-learn [8], Shap [9], XGBoost [10], InterpretML [11], Plotly, UMAP [12], pandas-profiling, mRMR [1], Lifelines [13] and SciPy [14].

Supplementary Table 1 MRI scanning protocol parameters

MRI sequence	Detailed parameters
T2w sequences	Pelvis: Matrix size: 235x512, in-plane resolution: 1.1x0.8x5mm; FoV: 262x400mm; TR: 5650ms; TE: 105ms. Prostate: Matrix size: 346x384, in-plane resolution: 0.6x0.5x3mm; FoV: 200x200mm; TR: 4000ms; TE: 104ms.
T2w 3D SPACE for MPR-reconstructions	Matrix size: 289x320, in-plane resolution: 0.9x0.9x0.9mm; FoV: 268x300mm; TR: 1800ms; TE: 128ms. WB (incl. T1-images)
T2w HASTE	Matrix size: 256x256, in-plane resolution: 1.56x1.5x6mm; FoV: 380x380mm; TR: 1400ms; TE: 121ms.
T1 VIBE fs post KM	Matrix size: 195x320, in-plane resolution: 1.6x1.2x3mm; FoV: 309x380mm; TR: 4.56ms; TE: 2.03ms.
DCE-VIBE sequences	Matrix: 138x192; in-plane resolution: 1.9x1.4x3.6; FoV: 260x260mm
DWI sequences	Matrix size: 102x160, in-plane resolution: 2.2x1.6x3.6mm; FoV: 260x221mm; b-values: 0,100, 850; TR: 5400ms; TE: 93ms.

Supplementary Table 2 Machine learning parameters

Section	Setting	Value
Preprocessing	max_missing_ratio	0.3
	number_of_selected_features	sqrtn
	imputation_method	knn
EDA	perform_reporting	TRUE
	perform_umap	TRUE
	perform_tsne	TRUE
	perform_pca	TRUE
Calibration	perform_calibration	FALSE
Training	number_of_folds	100
	test_set_ratio	0.2
	randomizedsearchcv_cv	5
	randomizedsearchcv_n_iter	10
Classifiers	classifiers_to_run	rf, xgb, dt, lgr, ebm
XAI	perform_permutation_importance	TRUE
	perform_shap	TRUE
	perform_surrogate_modeling	TRUE
	perform_partial_dependence_plotting	TRUE
Plotting	plot_roc_curves	TRUE
	show_fold_wise_roc	FALSE
EBM_hyperparameters	feature_names	None
	feature_types	None
	max_bins	256
	max_interaction_bins	64
	binning	quantile
	mains	all
	interactions	5
	outer_bags	8, 16
	inner_bags	0, 8
	learning_rate	0.01, 0.001, 0.0001
	validation_size	0.15
	early_stopping_rounds	50
	early_stopping_tolerance	0.0001
	max_rounds	10
	min_samples_leaf	2, 4
	max_leaves	3
	n_jobs	-2
	random_state	0
KNN_hyperparameters	weights	distance

	algorithm	auto
	leaf_size	30
	p	1, 2, 3, 4, 5
	metric	minkowski
	metric_params	None
	n_jobs	-1
DT_hyperparameters	criterion	gini
	splitter	best, random
	max_depth	1, 2, 3, 4, 5, 7, 10, 15
	min_samples_split	2, 4, 2006
	min_samples_leaf	1, 3, 2005
	min_weight_fraction_leaf	0
	max_features	
	max_features	auto, sqrt, log2
	random_state	0
	max_leaf_nodes	None
	min_impurity_decrease	0
	class_weight	None
	ccp_alpha	0
NN_hyperparameters	hidden_layer_sizes	(32, 64, 32)
	activation	relu, tanh, logistic
	solver	adam
	alpha	0.0001
	batch_size	auto
	learning_rate	constant
	learning_rate_init	0.01, 0.001, 0.0001
	power_t	0.5
	max_iter	1000
	shuffle	TRUE
	random_state	0
	tol	0.0001
	verbose	FALSE
	warm_start	FALSE
	momentum	0.9
	nesterovs_momentum	TRUE
	early_stopping	TRUE
	validation_fraction	0.1
	beta_1	0.9
	beta_2	0.999

	epsilon	1.00E-08
	n_iter_no_change	20
	max_fun	15000
RF_hyperparameters	n_estimators	100
	criterion	entropy
	max_depth	2, 4, 5, 10, 15
	min_samples_split	2, 4, 2008
	min_samples_leaf	1, 3, 5, 7
	min_weight_fraction_leaf	0
	max_features	auto, sqrt, log2
	max_leaf_nodes	None
	min_impurity_decrease	0
	bootstrap	TRUE
	oob_score	FALSE
	n_jobs	-1
	random_state	0
	verbose	0
	warm_start	FALSE
	class_weight	None
	ccp_alpha	0
	max_samples	None
XGB_hyperparameters	objective	binary:logistic
	use_label_encoder	FALSE
	base_score	None
	booster	None
	callbacks	None
	colsample_bylevel	None
	colsample_bynode	None
	colsample_bytree	0.5, 0.7, 1
	early_stopping_rounds	None
	enable_categorical	FALSE
	eval_metric	None
	gamma	0, 0.2
	gpu_id	None
	grow_policy	None
	importance_type	None
	interaction_constraints	None
	learning_rate	0.2, 0.3
	max_bin	None

	max_cat_to_onehot	None
	max_delta_step	None
	max_depth	2, 4, 6, 8
	max_leaves	None
	min_child_weight	1, 3
	missing	nan
	monotone_constraints	None
	n_estimators	100
	n_jobs	-1
	num_parallel_tree	None
	predictor	None
	random_state	0
	reg_alpha	None
	reg_lambda	None
	sampling_method	None
	scale_pos_weight	None
	subsample	None
	tree_method	None
	validate_parameters	None
	verbosity	None
SVM_hyperparameters	C	0.1, 1.0, 10
	kernel	rbf, poly, sigmoid, linear
	degree	2, 3, 2004
	gamma	scale, auto
	coef0	0
	shrinking	TRUE
	probability	TRUE
	tol	0.001
	cache_size	200
	class_weight	None
	verbose	FALSE
	max_iter	-1
	decision_function_shape	ovr
	break_ties	FALSE
	random_state	0
LGR_hyperparameters	penalty	l2
	dual	FALSE
	tol	0.0001, 0.001, 0.00001
	C	0.1, 1, 10

	fit_intercept	TRUE
	intercept_scaling	1
	class_weight	None
	random_state	None
	solver	lbfgs, liblinear
	max_iter	100
	multi_class	auto
	verbose	0
	warm_start	FALSE
	n_jobs	None
	l1_ratio	None

Supplementary Table 3 Clinical characteristics of the independent validation cohort.

Feature	Feature value	Entire data	No EPE	EPE	*p* value
Number of patients, n (%)		30 (100.00%)	15 (50.00%)	15 (50.00%)	
ISUP	1	0 (0%)	0 (0%)	0 (0%)	0.00658
	2	11 (36.67%)	10 (66.67%)	1 (6.67%)	
	3	2 (6.67%)	1 (6.67%)	1 (6.67%)	
	4	10 (33.33%)	2 (13.33%)	8 (53.33%)	
	5	7 (23.33%)	2 (13.33%)	5 (33.33%)	
Bone metastases (PET)	0	27 (90.0%)	14 (93.33%)	13 (86.67%)	1
	1	3 (10.0%)	1 (6.67%)	2 (13.33%)	
Nodule status (PET)	0	23 (76.67%)	10 (66.67%)	13 (86.67%)	1
	1	3 (10.0%)	1 (6.67%)	2 (13.33%)	
	NA	4 (13.33%)	4 (26.67%)	0 (0%)	
Metastases (PET)	0	12 (40.0%)	3 (20.0%)	9 (60.0%)	0.50549
	1	2 (6.67%)	1 (6.67%)	1 (6.67%)	
	NA	16 (53.33%)	11 (73.33%)	5 (33.33%)	
Tumor stage (PET)	1	5 (16.67%)	2 (13.33%)	3 (20.0%)	0.71338
	3	3 (10.0%)	1 (6.67%)	2 (13.33%)	
	4	1 (3.33%)	0 (0%)	1 (6.67%)	
	5	2 (6.67%)	0 (0%)	2 (13.33%)	
	6	2 (6.67%)	0 (0%)	2 (13.33%)	
	7	1 (3.33%)	0 (0%)	1 (6.67%)	
	NA	16 (53.33%)	12 (80.0%)	4 (26.67%)	
Seminal vesicle invasion (PET)	0	11 (36.67%)	3 (20.0%)	8 (53.33%)	1
	1	3 (10.0%)	0 (0%)	3 (20.0%)	
	NA	16 (53.33%)	12 (80.0%)	4 (26.67%)	
Ratio positive cylinders		0.54 (0.25)	0.52 (0.18)	0.57 (0.29)	0.54034
Age		65.83 (8.01)	64.87 (8.88)	66.8 (6.89)	0.50568
BMI		25.94 (3.43)	26.82 (2.05)	25.06 (4.21)	0.08802
Preoperative PSA		14.79 (15.62)	13.06 (15.93)	16.52 (15.11)	0.20579

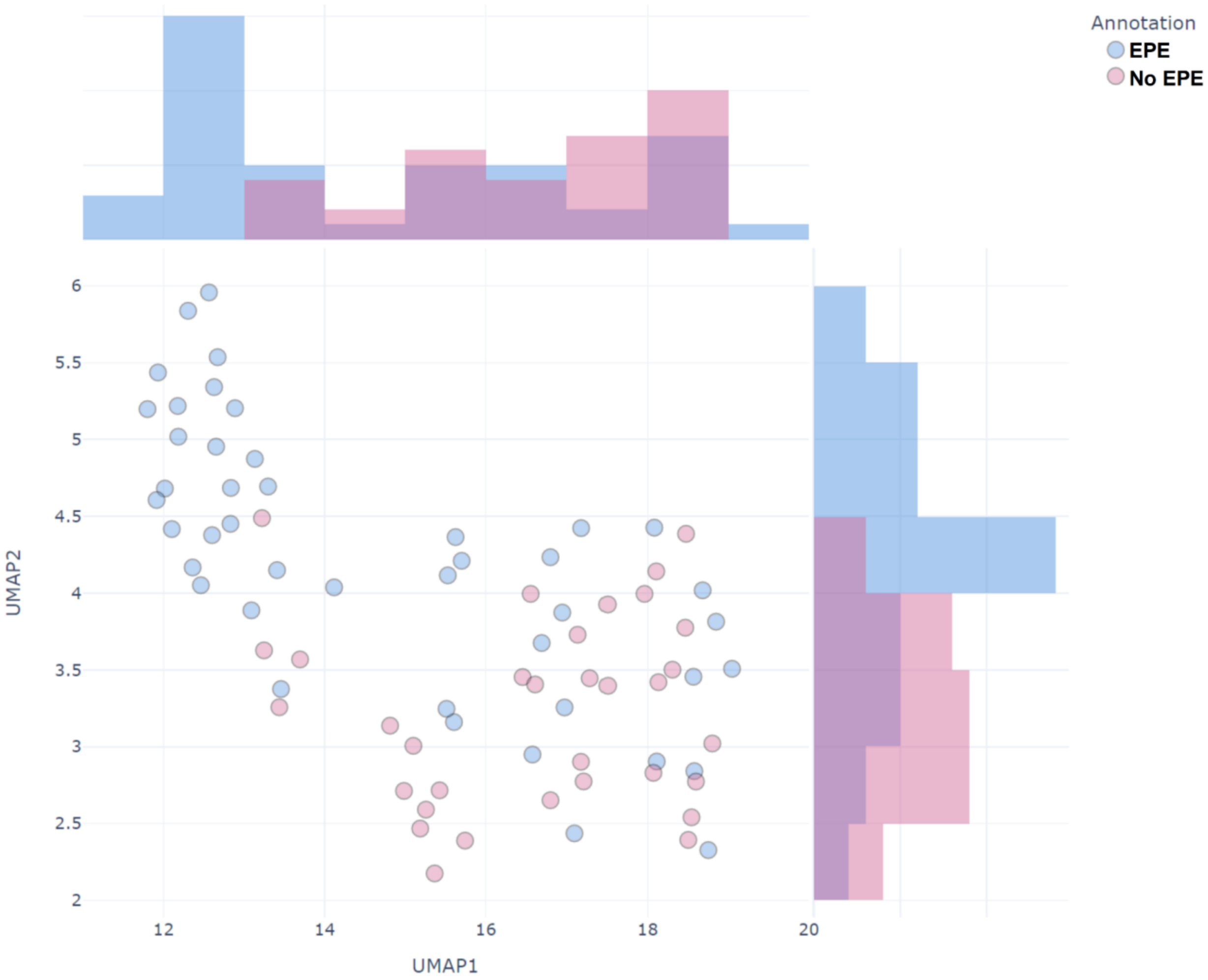

Supplementary Fig 1 UMAP visualization of patient similarity over all invasive and non-invasive features. Each dot represents a patient. Closer points indicate a higher similarity over all features

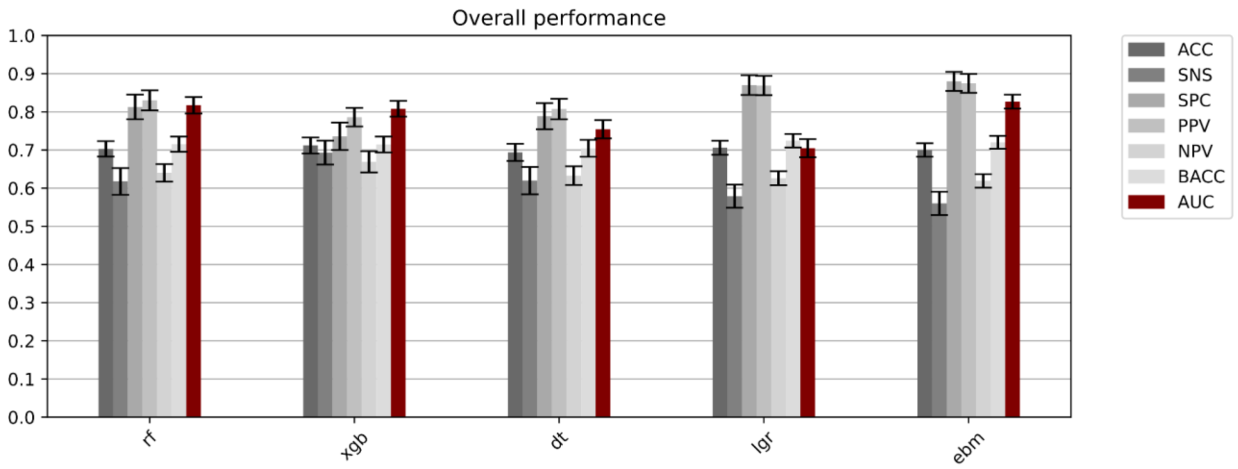

Supplementary Fig 2 Performance for ML model with non-invasive features only. ACC=Accuracy. SNS=Sensitivity. SPC=Specificity. PPV=Positive predictive value. NPV=Negative predictive. BACC=Balanced accuracy. AUC=Area under the receiver operating characteristic curve. rf=Random forest. xgb=Extreme gradient boosting. dt=Decision tree. lgr=Logistic regression. ebm=Explainable boosting machine

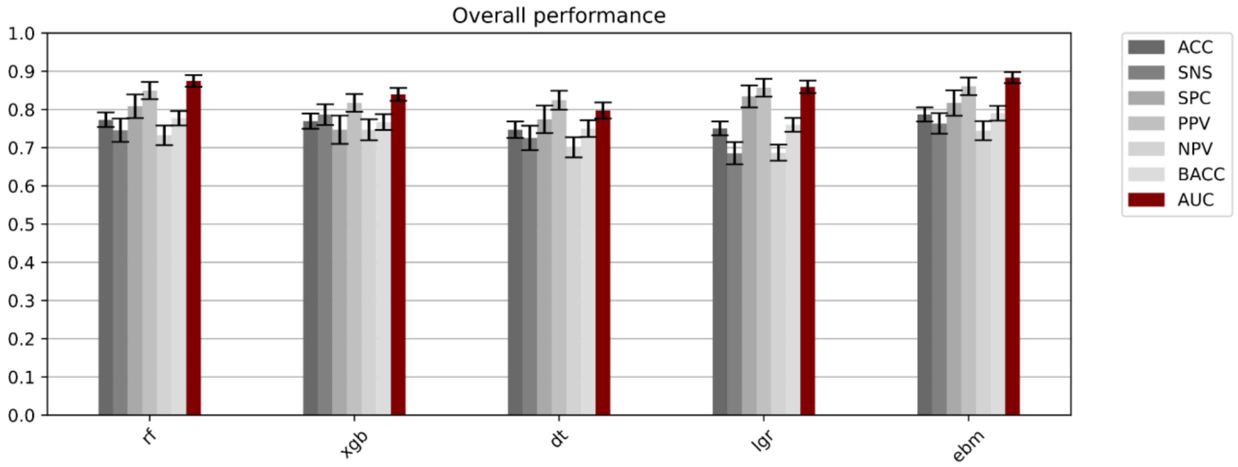

Supplementary Fig 3 Performance for ML model with invasive and non-invasive features. ACC=Accuracy. SNS=Sensitivity. SPC=Specificity. PPV=Positive predictive value. NPV=Negative predictive. BACC=Balanced accuracy. AUC=Area under the receiver operating characteristic curve. rf=Random forest. xgb=Extreme gradient boosting. dt=Decision tree. lgr=Logistic regression. ebm=Explainable boosting machine

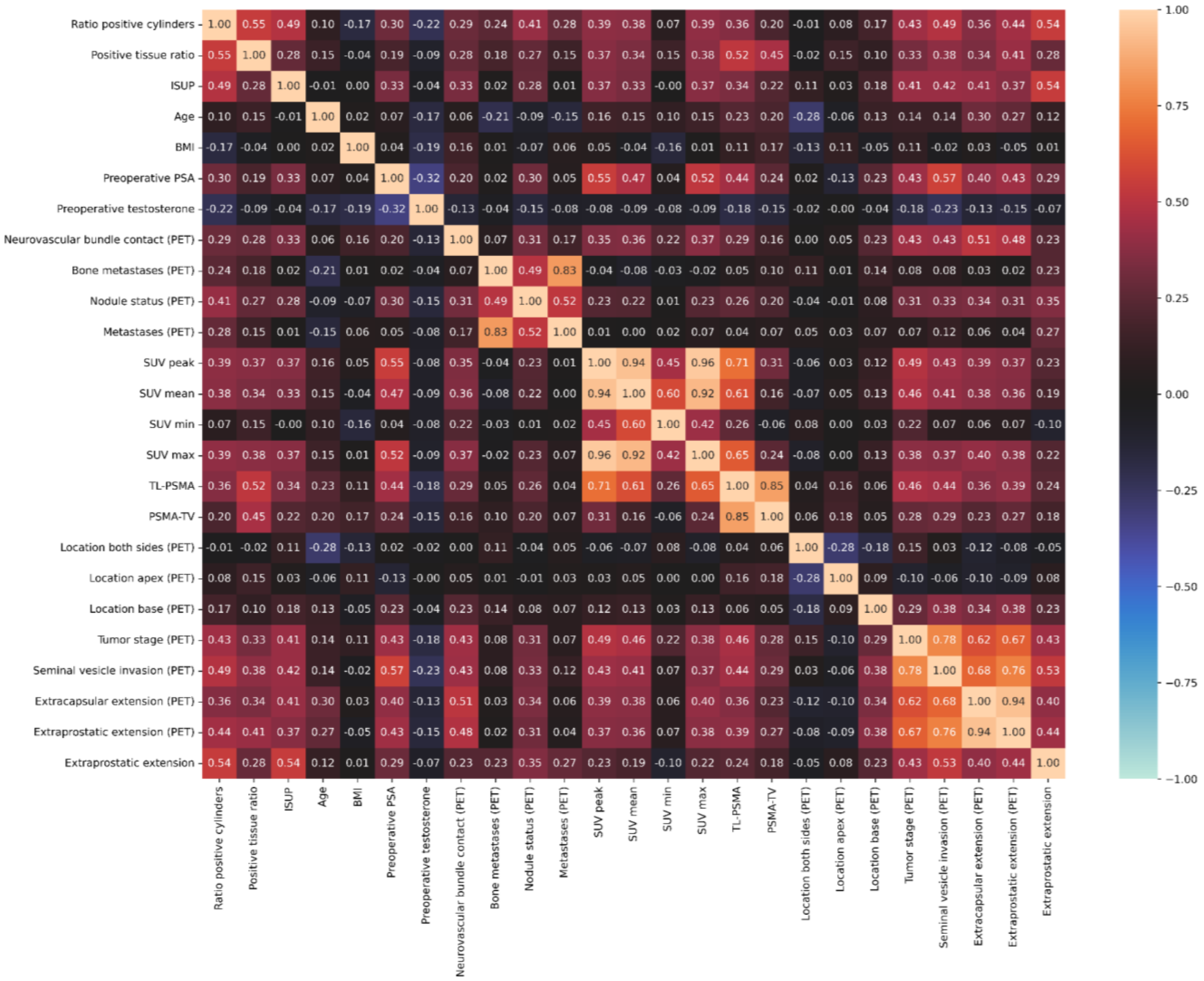

Supplementary Fig. 4 Correlation matrix of invasive and non-invasive features and outcome

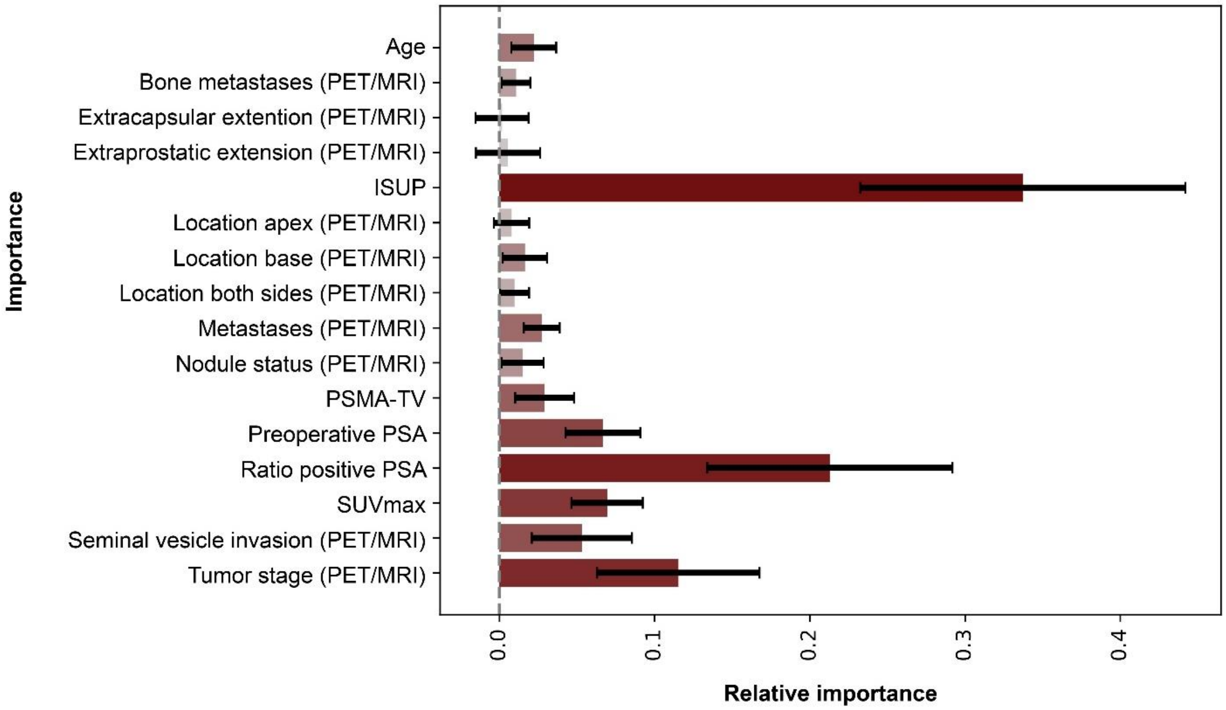

Supplementary Fig. 5 Permutation importance for the EBM model including invasive and non-invasive features

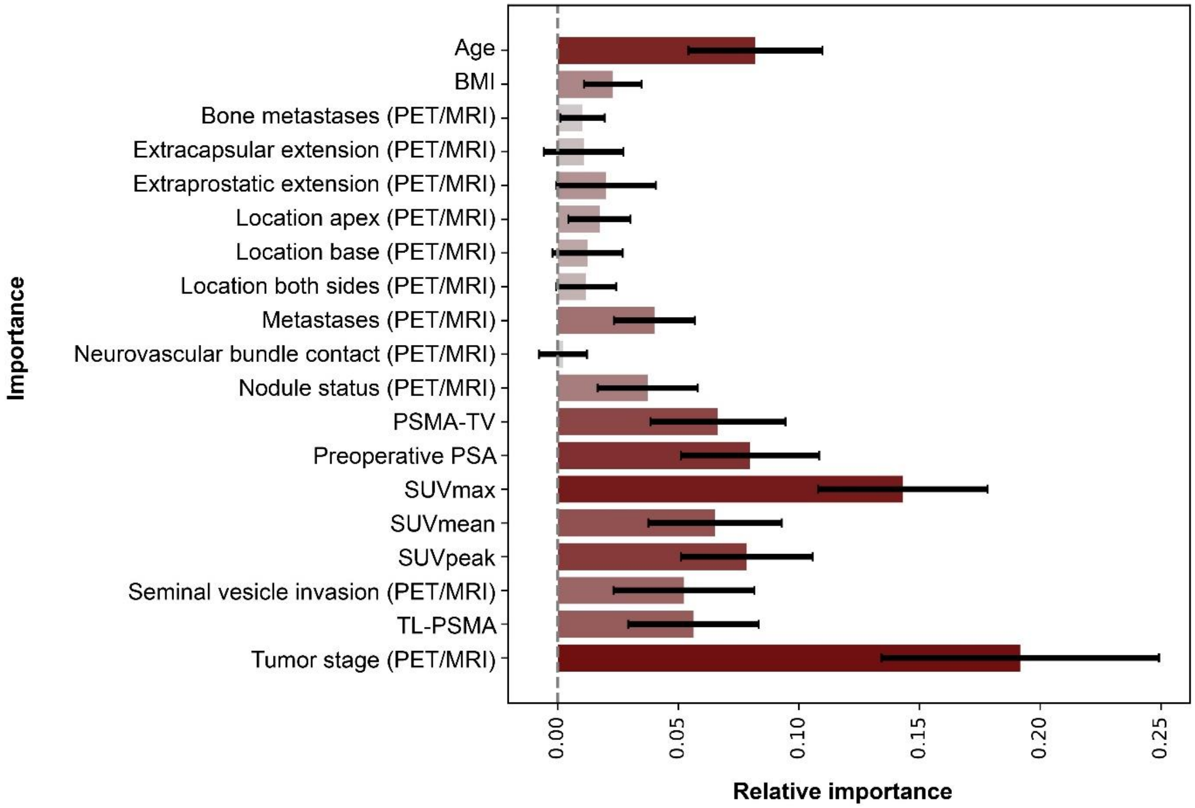

Supplementary Fig. 6 Permutation importance for the EBM model including non-invasive features only

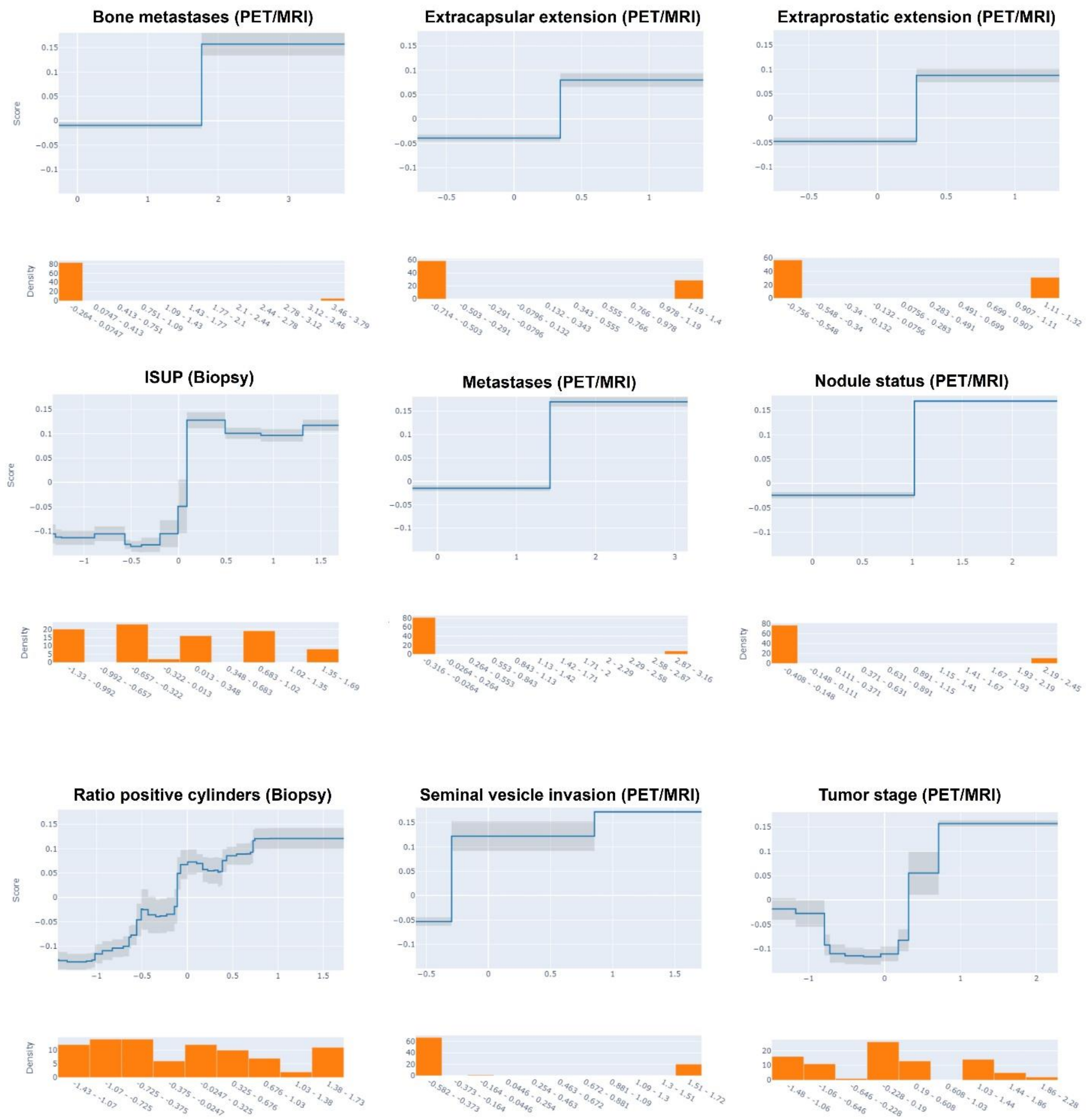

Supplemetal Fig. 7 Explainable-boosting machine-specific partial dependence plots indicating the impact between precise feature values on the model output. For the line plots, the x-axis indicates the normalized feature value while the y-axis indicates the impact on the model output. Higher output values lead to a higher probability for the prediction of EPE in the overall model. The corresponding histograms indicate the number of patients (y-axis) associated with a given feature range (x-axis). The number of patients at a given range is a surrogate indicator for the model's robustness when a feature at the given value is used in the model. Due to feature imputation, patients with impossible values (e.g. ISUP grade 2.5) may occur in the histograms

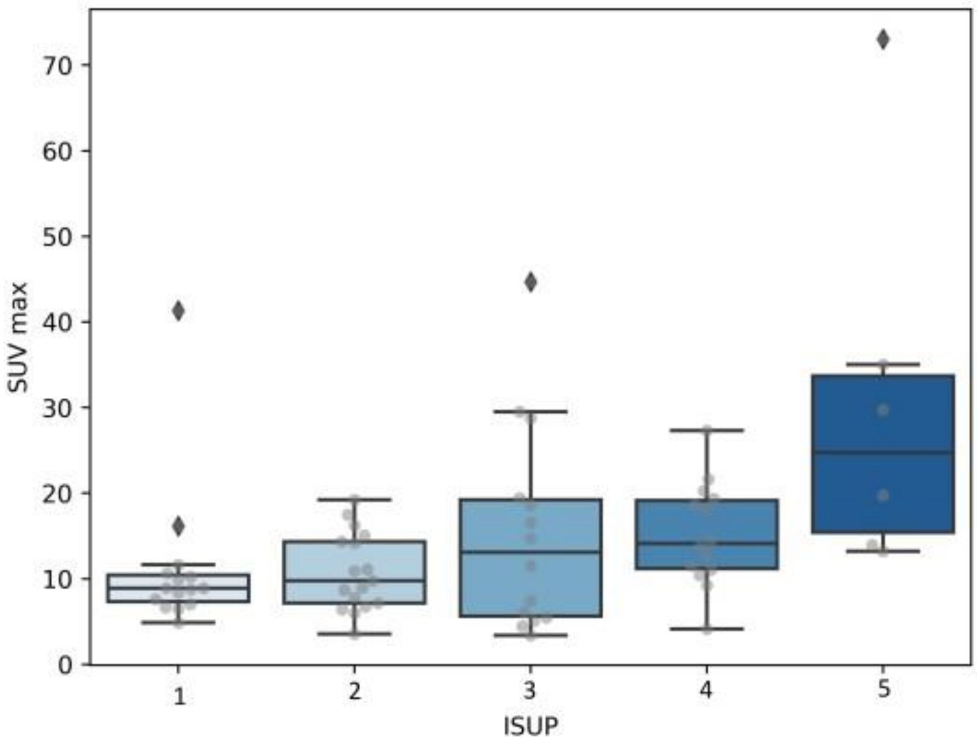

Supplemental Fig. 8 Association of ISUP and SUVmax. Spearman rank correlation indicated a slight but highly significant positive correlation coefficient of 0.37 (p < 0.001). Median SUVmax was consistently increasing for each higher ISUP group. ISUP=International Society of Urological Pathology (grade)

Performance metrics

True positive (TP): Test result that was correctly predicted as positive

True negative (TN): Test result that was correctly predicted as negative

False positive (FP): Test result that was incorrectly predicted as positive

False negative (FN): Test result that was incorrectly predicted as negative

Area under the receiver operating characteristic curve (AUC): AUC was calculated via scikit-learn version 1.1.0[15].

Accuracy (ACC):

$$ACC = \frac{\mathrm{TP} + \mathrm{TN}}{\mathrm{TP} + \mathrm{TN} + \mathrm{FP} + \mathrm{FN}}$$

Sensitivity (SNS):

$$SNS = \frac{\mathrm{TP}}{\mathrm{TP} + \mathrm{FN}}$$

Positive predictive value (PPV):

$$PPV = \frac{\mathrm{TP}}{\mathrm{TP} + \mathrm{FP}}$$

Specificity (SPC):

$$SPC = \frac{\mathrm{TN}}{\mathrm{TN} + \mathrm{FP}}$$

Negative predictive value (NPV):

$$NPV = \frac{\mathrm{TN}}{\mathrm{TN} + \mathrm{FN}}$$

References1. Peng H, Long F, Ding C. Feature selection based on mutual information: criteria of max-dependency, max-relevance, and min-redundancy. IEEE Trans Pattern Anal Mach Intell. 2005;27:1226–38.

2. Chawla NV, Bowyer KW, Hall LO, Kegelmeyer WP. SMOTE: Synthetic Minority Over-sampling Technique. jair. 2002;16:321–57.

3. Harris CR, Millman KJ, van der Walt SJ, Gommers R, Virtanen P, Cournapeau D, et al. Array programming with NumPy. Nature. 2020;585:357–62.

4. Mckinney W. Pandas: A foundational Python library for data analysis and statistics [Internet]. dlr.de; 2011 [cited 2022 Oct 25]. Available from: https://www.dlr.de/sc/portaldata/15/resources/dokumente/pyhpc2011/submissions/pyhpc2011_submission_9.pdf

5. Waskom M. seaborn: statistical data visualization. J Open Source Softw. 2021;6:3021.

6. Hunter. Matplotlib: A 2D Graphics Environment. Comput Sci Eng. 2007;9:90–5.

7. Pedregosa F, Varoquaux G, Gramfort A, Michel V, Thirion B, Grisel O, et al. Scikit-learn: Machine Learning in Python [Internet]. arXiv [cs.LG]. 2012 [cited 2022 Oct 25]. Available from: https://www.jmlr.org/papers/volume12/pedregosa11a/pedregosa11a.pdf?ref=https://githubhelp.com

8. Lemaitre G, Nogueira F, Aridas CK. Imbalanced-learn: A python toolbox to tackle the curse of imbalanced datasets in machine learning [Internet]. arXiv [cs.LG]. 2016 [cited 2023 Jan 27]. Available from: https://www.jmlr.org/papers/volume18/16-365/16-365.pdf

9. Lundberg SM, Erion G, Chen H, DeGrave A, Prutkin JM, Nair B, et al. From Local Explanations to Global Understanding with Explainable AI for Trees. Nat Mach Intell. 2020;2:56–67.

10. Chen T, Guestrin C. XGBoost: A Scalable Tree Boosting System. Proceedings of the 22nd ACM SIGKDD International Conference on Knowledge Discovery and Data Mining. New York, NY, USA: Association for Computing Machinery; 2016. p. 785–94.

11. Nori H, Jenkins S, Koch P, Caruana R. InterpretML: A Unified Framework for Machine Learning Interpretability [Internet]. arXiv [cs.LG]. 2019. Available from: http://arxiv.org/abs/1909.09223

12. Becht E, McInnes L, Healy J, Dutertre C-A, Kwok IWH, Ng LG, et al. Dimensionality reduction for visualizing single-cell data using UMAP. Nat Biotechnol [Internet]. 2018; Available from: http://dx.doi.org/10.1038/nbt.4314

13. Davidson-Pilon C. lifelines: survival analysis in Python. J Open Source Softw. 2019;4:1317.

14. Virtanen P, Gommers R, Oliphant TE, Haberland M, Reddy T, Cournapeau D, et al. SciPy 1.0: fundamental algorithms for scientific computing in Python. Nat Methods. 2020;17:261–72.

15. Hand DJ, Till RJ. A Simple Generalisation of the Area Under the ROC Curve for Multiple Class Classification Problems. Mach Learn. 2001;45:171–86.
